# Risk factors and risk nomogram model of reoperation for hemorrhages after severe traumatic brain injury craniotomy

**DOI:** 10.1002/ibra.12032

**Published:** 2022-04-13

**Authors:** Tao Yang, Jie Yu, Hao Shen, Chao‐Zhi Yang, Ping Zhang, Yi Li, Hai‐Tao Wu

**Affiliations:** ^1^ Department of Neurosurgery Affiliated Hospital of Zunyi Medical University Zunyi Guizhou China

**Keywords:** nomogram model, postoperative hemorrhages, reoperation, risk factors, severe traumatic brain injury

## Abstract

**Objective:**

This study aimed to explore the risk factors associated with reoperation for postoperative hemorrhages after severe traumatic brain injury (sTBI) craniotomy and establish a risk nomogram model.

**Methods:**

A retrospective case‐control study was performed. Overall, 367 patients who were diagnosed with sTBI and fulfilled the inclusion criteria were enrolled from the Department of Neurosurgery of the Affiliated Hospital of Zunyi Medical University between January 2015 and December 2020. They were divided into a reoperation group and a non‐reoperation group according to whether they underwent reoperation for hemorrhages. Using univariate binary logistic regression analysis, the possible risk factors were screened. Subsequently, the independent risk factors of reoperation for postoperative hemorrhages were screened using the forward step method of multivariate binary logistic regression analysis, and a corresponding nomogram model was constructed. The receiver operative characteristic (ROC) curve was used to evaluate the reliability of the model. Finally, 30% of the data were randomly selected for internal verification of the model.

**Results:**

The reoperation rate for hemorrhage after sTBI emergency craniotomy was 14.71% (54/367); multivariate logistic regression analysis showed that multiple hemorrhages (odds ratio [OR] = 4.38, 95% confidence interval [CI]: 1.815–10.587, *p* = 0.001), day or night surgery (OR = 0.26, 95% CI: 0.119–0.547, *p* < 0.001), operation duration (OR = 0.74, 95% CI: 0.119–0.547, *p* < 0.025), and abnormal intraoperative blood pressure fluctuation (OR = 4.15, 95% CI: 2.090–8.245, *p* < 0.001) were statistically significant. The sensitivity and specificity of the nomogram model were 0.815 and 0.661, respectively, and the area under ROC curve was 0.76 (95% CI: 0.705–0.833). Internal verification showed that the area under the ROC curve was 0.783 (95% CI: 0.683–0.883).

**Conclusions:**

Taken together, the results of our study reveal that multiple preoperative intracranial hemorrhages, day and night operation, operation duration, and abnormal fluctuation of intraoperative blood pressure were independent risk factors for postoperative bleeding and reoperation for sTBI. Through the analysis of the influencing factors, a prediction model for the risk of bleeding and reoperation after craniocerebral trauma was developed. Compared with other relevant studies, this prediction model has good prediction efficiency and can be used to predict the occurrence of bleeding and reoperation after sTBI in patients.

## Graphical abstract

This paper confirmed the risk nomogram model has a good predictive effect by analyzing the factors related to hemorrhage reoperation after craniotomy for severe craniocerebral trauma and validate the internal data of the model. It is helpful for neurosurgeons to make a preoperative prediction and reduce the incidence of postoperative hemorrhages and reoperation.

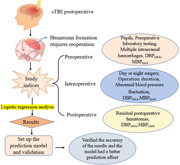

## 1 INTRODUCTION

Severe traumatic brain injury (sTBI) is a traumatic brain injury (TBI) with a Glasgow Coma Scale (GCS) score of ≤8 upon hospital admission; its incidence is 47.3–694 cases/100,000 individuals and the mortality rate accounts for 35%–42% of TBI patients.^1^, ^2^ A craniotomy for the evacuation of intracranial hematomas is an important treatment method for sTBI, and timely and effective surgical intervention can save the lives of patients and improve patient prognosis considerably; however, the surgical treatment outcome of sTBI is dependent on multiple factors, and there are many complications associated with the operation,^3^ with postoperative hemorrhages being one of the most common and serious complications after an sTBI craniotomy. Postoperative hemorrhages can occur at any location (including the surgical site) inside the skull in many forms. Hematomas cause increased intracranial pressure (ICP), and can again change the mental status and pupils; even postoperative hemorrhages can become life‐threatening and an emergency surgery has to be performed again to remove the hematomas. In addition, postoperative hemorrhages affect patient prognosis, affect the patient's mental and physical states, increase the financial burden on the patient and their family, reduce patient satisfaction, weaken doctors’ confidence, and even cause medical disputes. This study discusses the independent factors influencing reoperation for hemorrhages after sTBI and establishes a risk nomogram model for reoperation of hemorrhages after sTBI based on the discussed factors, with the aim of guiding individualized doctor–patient communication before surgery and developing targeted measures to prevent the occurrence of reoperation because of postoperative hemorrhages.

## 2 DATA AND METHODS

### 2.1 Research objects

A total of 367 patients with sTBI who underwent a craniotomy at the Department of Neurosurgery of the Affiliated Hospital of Zunyi Medical University from January 2015 to December 2020 were included.

The inclusion criteria were as follows: Patients with sTBI and admitted to the same hospital; patients in whom the first operation was an urgent craniotomy (or decompressive craniectomy) for the evacuation of hematomas; and patients with complete case data.

The exclusion criteria were as follows: Patients who had undergone craniotomy at other hospitals; patients for whom no craniocerebral computed tomography (CT) was performed after craniotomy; patients who were discharged from the hospital before 1 week; patients had undergone reoperation for any reason other than postoperative hemorrhages, such as postoperative infection and hydrocephalus; and patients in whom a comorbid large infarct was confirmed through preoperative cranial CT or during surgery.

Grouping design: A total of 367 patients were divided into a case group and a control group depending on whether or not a reoperation for hemorrhages was performed.

### 2.2 Indications for reoperation for postoperative hemorrhages

The following were the indications for reoperation for postoperative hemorrhages. After craniotomy, new intracranial hematomas were found through cranial CT. The hematomas had a significant mass effect and showed indications for emergency surgery: supratentorial hematoma >30 ml, subtentorial hematoma >10 ml, and significantly increased ICP; the clinical symptoms of patients deteriorated; there were other conditions for which the clinicians believed an emergency surgery should be performed for the evacuation of hematomas. All imaging data were separately analyzed by an associate chief physician at the Department of Neurosurgery and an associate chief physician at the Department of Radiology and then combined with the clinical manifestations of the patients to decide whether a reoperation should be performed.

### 2.3 Study indices

The case data of patients were checked and recorded. General indices were as follows: age, sex, history of hypertension, and duration from injury to surgery. Preoperative indices included the following: preoperative pupils, maximum systolic blood pressure (MBP_MAX_) before surgery, maximum diastolic blood pressure (DBP_MAX_) before surgery, preoperative laboratory testing (INR, PT, APTT, fibrinogen, and platelet count), and preoperative imaging examination (contralateral skull fracture, contralateral cerebral contusion and laceration, multiple intracranial hemorrhages [ICH], and midline shift). Intraoperative indices were as follows: day or night surgery, operation duration, surgeon title, bone flap decompression, intraoperative encephalocele, intraoperative blood transfusion, abnormal blood pressure fluctuation, intraoperative MBP_MAX_, and intraoperative DBP_MAX_. Finally, postoperative indices were postoperative MBP_MAX_, postoperative DBP_MAX_, and residual postoperative hematomas.

### 2.4 Statistical analysis

In this study, we used SPSS 24 (statistical software) and Empower Stats 3.0 for data analysis. Continuous variables are expressed as mean ± standard deviation (*x̅* ± s), and categorical variables are expressed as a percentage (%). SPSS 24 (statistical software) and univariate binary logistic regression analysis were used to screen out the influencing factors that might result in the need for reoperation for postoperative hemorrhages, and then the forward step method of multivariate binary logistic regression analysis was used to screen out the independent influencing factors. Empower Stats 3.0 was used to construct the risk nomogram for predicting the reoperation for hemorrhages after sTBI surgery. Empower Stats software was used to randomly select 30% of the data in this study for the internal verification of the prediction model, and a receiver operating characteristic (ROC) curve was used to evaluate the prediction model. A *p* < 0.05 was considered to be statistically significant.

## 3 RESULTS

Of the 367 patients with sTBI, 286 were males and 81 were females, aged between 1 and 87 years (average age, 42.45 years). Preoperative craniocerebral CT indicated that 261 patients had multiple hemorrhages and 106 patients did not have multiple hemorrhages; in addition, 49 patients underwent surgery in the daytime and 318 patients underwent surgery in the night time and 73 patients had abnormal fluctuations of blood pressure during the operation, whereas the remaining 294 patients did not. Also, 54 patients underwent reoperation for postoperative hemorrhages, and 313 patients did not suffer from postoperative hemorrhages.

Univariate binary logistic analysis was conducted for study indices, and the results showed that the factors influencing reoperation for postoperative hemorrhages of patients with sTBI included age, preoperative MBP_MAX_, multiple hemorrhages before primary surgery, day or night surgery, surgeon title, intraoperative MBP_MAX_, abnormal intraoperative blood pressure fluctuations, residual postoperative hematomas, and operation duration. For those possible factors with *p* < 0.1 in univariate analysis, multivariate binary logistic regression analysis was performed, which indicated the following factors as independent risk factors for reoperation for postoperative hemorrhages (Table 1): preoperative multiple hemorrhages, day or night surgery, operation duration, and abnormal blood pressure fluctuation.

**Table 1:** Univariate analysis and multivariate analysis of the related factors of reoperation for hemorrhages after sTBI surgery

Selected variables	Univariate analysis	Multivariate analysis
	OR (95% CI); *p* Value	OR (95% CI); *p* Value
Age	1.020 (1.003, 1.037); **0.022**	1.010 (0.991, 1.030); 0.297
Preoperative MBP_MAX_	1.014 (1.004, 1.024); **0.004**	1.010 (0.993, 1.027); 0.238
Multiple hemorrhages	3.106 (1.356, 7.116); **0.007**	4.383 (1.815, 10.587); **<0.01**
Day or night surgery	0.317 (0.158, 0.634); **0.001**	0.255 (0.119, 0.547); **<0.01**
Operation duration	0.083 (0.632, 1.029); **0.083**	0.740 (0.569, 0.963); **0.025**
Surgeon title		
Chief physician	0.045	0.135
Associate chief physician	0.72 (0.007, 0.730); **0.026**	0.152 (0.013, 1.823); 0.137
Physician‐in‐charge	0.047 (0.005, 0.464); **0.009**	0.091 (0.008, 1.062); 0.056
Resident physician	0.067 (0.004, 1.017); **0.051**	0.168 (0.009, 3.093); 0.168
Abnormal blood pressure fluctuation	2.886 (1.543, 5.398); **0.001**	4.151 (2.090, 8.245); **<0.01**
Maximum systolic blood pressure	1.015 (0.999, 1.031); **0.058**	0.995 (0.974, 1.017); 0.658
Residual hematomas or not	1.903 (1.028, 3.522); **0.041**	1.671 (0.830, 3.365); 0.150

Abbreviations: CI, confidence interval; MBP_MAX_, maximum systolic blood pressure; OR, odds ratio; sTBI, severe traumatic brain injury.

**Figure 1:**
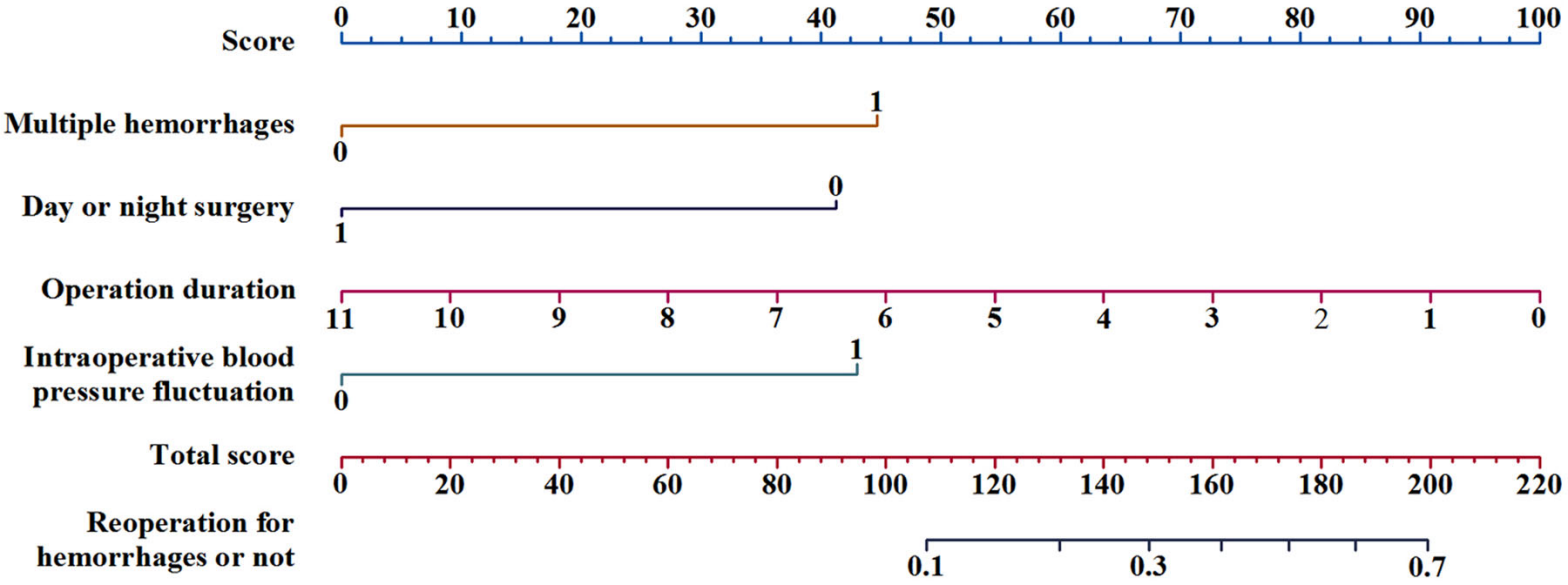
Risk prediction nomogram model of reoperation for hemorrhages after severe traumatic brain injury surgery [Color figure can be viewed at wileyonlinelibrary.com]

**Figure 2:**
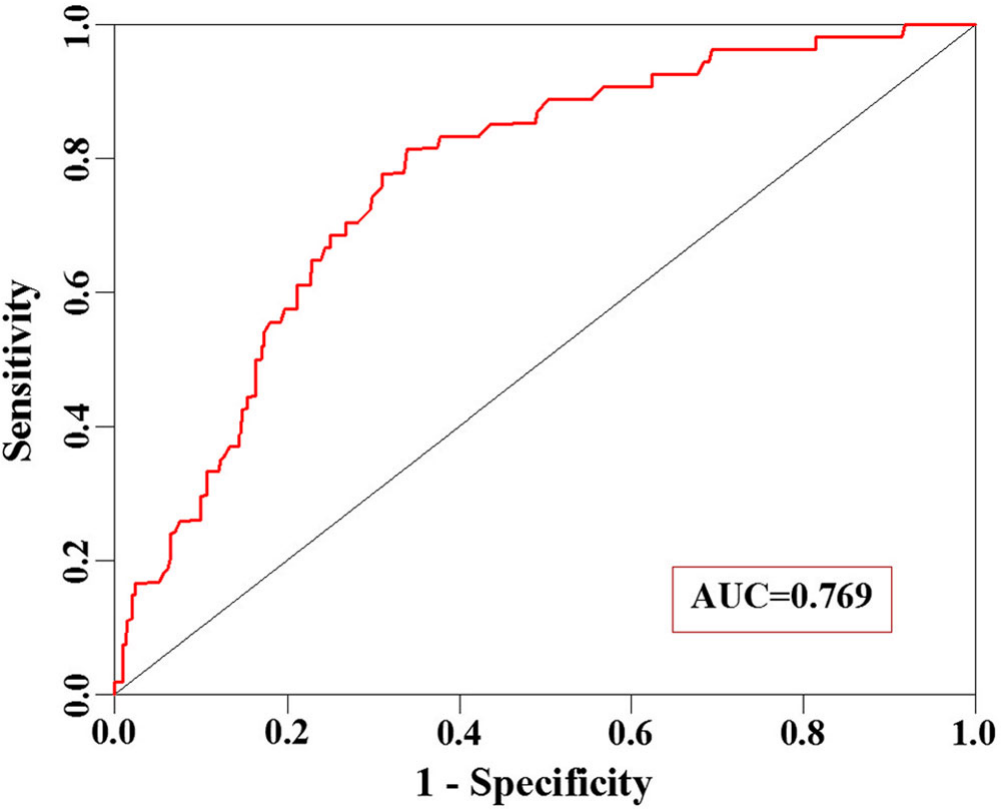
Receiver operating characteristic curve of the nomogram model of reoperation for hemorrhages after severe traumatic brain injury surgery (area under the curve [AUC]: 0.826, sensitivity: 77.6%, specificity: 80.6%) [Color figure can be viewed at wileyonlinelibrary.com]

**Figure 3:**
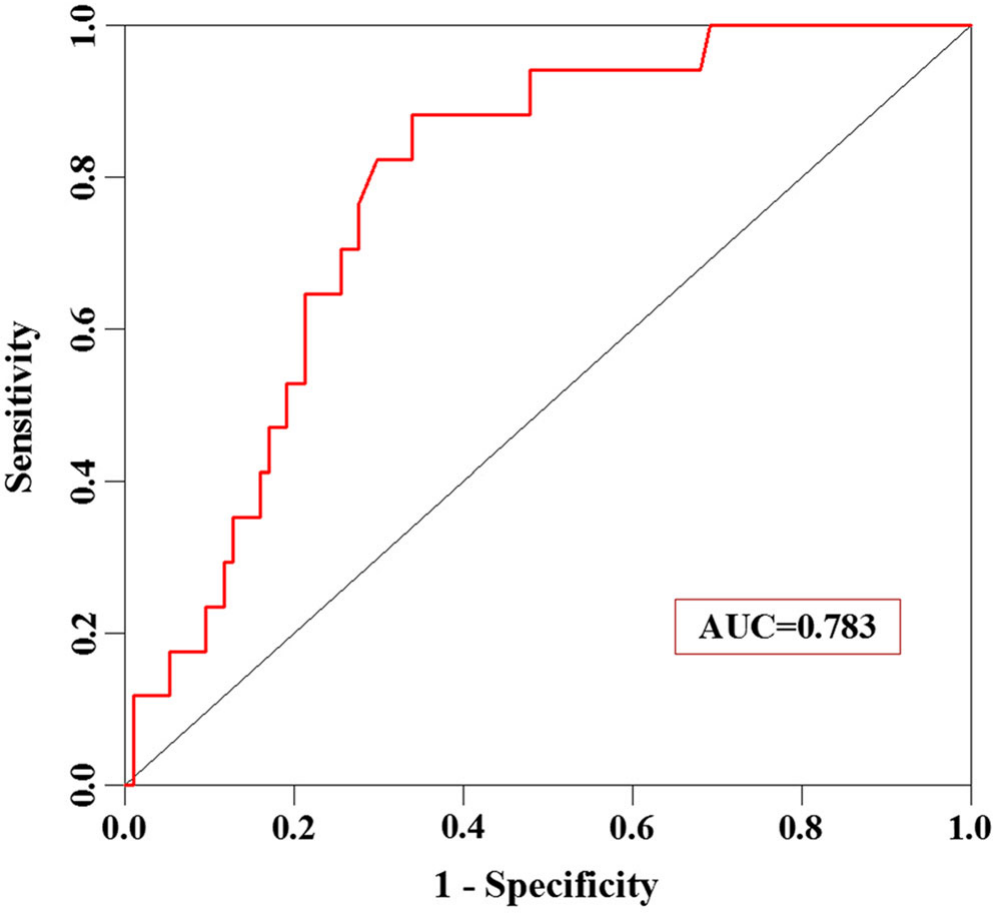
Receiver operating characteristic curve of the internal verification population of the model of reoperation for hemorrhages after severe traumatic brain injury surgery (area under the curve [AUC]: 0.869, sensitivity: 90.9%, specificity: 84.6%) [Color figure can be viewed at wileyonlinelibrary.com]

Based on these independent risk factors for reoperation for hemorrhage after sTBI surgery, we established a risk nomogram predicting reoperation for hemorrhages after sTBI surgery (Figure 1). A score was assigned to each of the four variables in each patient and the corresponding scores of each of these variables were added to calculate the total patient score, which was further used to determine the risk of reoperation for hemorrhages after sTBI surgery. The ROC curve was constructed to obtain the following values: the area under the curve (AUC) was 0.769, Youden's index was 0.476, the sensitivity was 81.5%, and the specificity was 66.1% (Figure 2). Furthermore, 30% of the samples used in modeling were used for the internal verification of the model. The results showed an AUC of 0.783 (Figure 3).

## 4 DISCUSSION

Postoperative hemorrhage reoperation is a serious postoperative complication in patients with craniocerebral trauma who need emergency craniotomy to remove intracranial hematoma because of various reasons within 2 weeks after craniotomy. Intracranial hematomas were confirmed via imaging examination, and emergency surgery was required to remove the hematomas. Accordingly, we concluded that the rate of reoperation for postoperative hemorrhages in patients with sTBI was approximately 14.7%, which was lower than the rate (16.2%) reported by Khalili et al.^4^ and also lower than the incidence (21.9%) of reoperation for postoperative hemorrhages at distant sites among patients who have undergone sTBI craniotomy in China.^5^ In this study, preoperative multiple hemorrhages, day or night surgery, operation duration, and abnormal blood pressure fluctuations were the independent influencing factors for reoperation for hemorrhages after sTBI surgery.

Multiple ICHs indicate that hemorrhages occur at two or more sites inside the skull, with a wider area of cerebral contusion. Lan et al.^6^ believed that a small amount of hemorrhage was an important prediction factor for reoperation for hemorrhages after sTBI surgery and that, before surgery, clinicians should be more alert toward those seemingly small and less obvious hemorrhage sites, indirectly confirming the relation between multiple ICHs and reoperation for hemorrhages after sTBI surgery. Mukerji et al.^7^ found that performing an emergency surgery at times other than between 8:00 a.m. and 5:00 p.m. was associated with unplanned postoperative reoperations, and the risk was 1.5 times as high as that for surgeries performed between 8:00 a.m. and 5:00 p.m. The results of our study indicated that an emergency surgery at night time (not between 8:00 a.m. and 6:00 p.m.) was a protective factor for reoperation for hemorrhages after sTBI surgery and the possible causes for this were as follows: when emergency surgery was performed at night time or round the clock, the surgeons were more careful and cautious during the surgery and more strictly carried out the “5‐min hemorrhage suspension test”; also, the nocturnal blood pressure in healthy individuals is lower than the blood pressure measured during the daytime. When the emergency surgery was performed at night time, the blood pressure did not fluctuate considerably during surgery because the blood pressure was affected by the adjustment from daytime to night time. Jian et al.^8^ had conducted a 1:1 matched case‐control study for those patients who underwent selective craniotomy at the Department of Neurosurgery and believed that the maximum systolic blood pressure (>160 mmHg) during and after surgery and the average maximum blood pressure (>110 mmHg) during and after surgery are risk factors for reoperation for hemorrhages after craniotomy. In our study findings, the maximum systolic blood pressure during surgery was not an influencing factor for reoperation for postoperative hemorrhages. If the intraoperative systolic blood pressure was higher than 160 mmHg and the blood pressure fluctuation between two points was greater than 20 mmHg, it was defined as an intraoperative blood pressure fluctuation. In this case, the impact of the intraoperative blood pressure fluctuation on reoperation for postoperative hemorrhages was studied and both univariate and multivariate regression analyses showed that intraoperative blood pressure fluctuation is independently associated with reoperation for hemorrhages after sTBI surgery.

The study of the prediction model of reoperation after the neurosurgical procedures included a prediction model of reoperation for hemorrhages after craniotomy for neurological diseases,^9^ a prediction model of reoperation after neurooncology surgery,^10^ and a prediction model of reoperation for recurrence after trepanation of chronic epidural hematoma.^11^ There are very few studies on a prediction model of reoperation for hemorrhages after sTBI surgery; therefore, the development of a reliable and effective prediction model of reoperation for hemorrhages after sTBI surgery is of great practical significance. Zhao et al.^12^ had studied the predictive factors for reoperation after sTBI surgery and conducted a ROC curve analysis for the prediction model. The results showed an AUC of 0.771. However, the study of Zhao et al. excluded patients with cerebellar contusion, sTBI, and coagulation disorders and individuals older than 65 years of age, that is, the scope of the study population limited the widespread clinical use of their model. In contrast, in our model, all patients with sTBI who fulfilled the inclusion and exclusion criteria were studied; all preoperative and intraoperative indices were included and the established prediction model had wider clinical applicability to reoperation for postoperative hemorrhages. The ROC curve analysis and internal verification of the model showed that the model that we established has good capability to identify the requirement of reoperation for hemorrhages after sTBI surgery.^13^

## 5 CONCLUSIONS

To sum up, this study concludes that multiple preoperative hemorrhages, day or night surgery, operation duration, and abnormal blood pressure fluctuation had an independent impact on reoperation for hemorrhages after sTBI surgery. The risk nomogram model of reoperation for hemorrhages after sTBI surgery was constructed through an analysis of influencing factors and it was confirmed that this model had a better prediction effect through internal data verification of the model. However, given that this is a single‐center retrospective clinical study and lacks external verification of the model, multicenter clinical studies based on large samples should be conducted further in later studies.

## AUTHOR CONTRIBUTIONS

Tao Yang and Jie Yu wrote the initial draft of the paper. Hao Shen and Chao‐Zhi Yang contributed the central idea and Ping Zhang reviewed the literature. Hai‐Tao Wu and Yi Li revised the paper.

## CONFLICTS OF INTEREST

The authors declare no conflicts of interest.

## ETHICS STATEMENT

This study was conducted ethically in accordance with the Declaration of Helsinki and approved by the Biomedical Research Ethics Committee of the Affiliated Hospital of Zunyi Medical University (No. KLLY‐2020‐017). The patients provided written consent to publish this case. Written consent was signed by all patients who were 18 years of age and older. For patients younger than 18 years of age, a legal guardian or parent signed the consent form.

## Data Availability

The data used to support the findings of this study are available from the corresponding author upon request.
